# Indexing of superimposed Laue diffraction patterns using a dictionary–branch–bound approach

**DOI:** 10.1107/S1600576722006021

**Published:** 2022-08-24

**Authors:** Anthony Seret, Wenqiang Gao, Dorte Juul Jensen, Andy Godfrey, Yubin Zhang

**Affiliations:** a Department of Civil and Mechanical Engineering, Technical University of Denmark, Kongens Lyngby, 2800, Denmark; b Key Laboratory of Advanced Materials, School of Materials Science and Engineering, Tsinghua University, Beijing, 100084, People’s Republic of China; Ecole National Supérieure des Mines, Saint-Etienne, France

**Keywords:** Laue diffraction, superimposed patterns, indexing, crystallographic orientations

## Abstract

X-ray Laue diffraction is an important method for characterizing the local crystallographic orientation and elastic strain in polycrystalline materials. Existing analysis methods are designed mainly to index a single or a few Laue diffraction pattern(s) recorded in a detector image. In this work, a novel method called dictionary–branch–bound (DBB) is presented to determine the crystallographic orientations of multiple crystals simultaneously illuminated by a parallel X-ray incident beam, using only the spot positions in a detector image. DBB is validated for simulated X-ray Laue diffraction data. In the simulation, up to 100 crystals with random crystallographic orientations are simultaneously illuminated. Fake spots are randomly added to the detector image to test the robustness of DBB. Additionally, spots are randomly removed to test the resilience of DBB against true spots that are undetected due to background noise and/or spot overlap. Poisson noise is also added to test the sensitivity of DBB to less accurate positions of detected spots. In all cases except the most challenging one, a perfect indexing with a mean angular error below 0.08° is obtained. To demonstrate the potential of DBB further, it is applied to synchrotron microdiffraction data. Finally, guidelines for using DBB in experimental data are provided.

## Abstract 2

A method is developed to determine multiple simultaneously illuminated crystallographic orientations producing superimposed Laue diffraction patterns on a single detector image, using only spot positions. Tests performed on simulated data show the capability of indexing at least 100 crystals, even if fake spots are added, true spots are removed and noise is added. Further validation on synchrotron microdiffraction data is also provided.

## 1. Introduction

Laue diffraction occurs when a parallel broad-bandpass X-ray beam illuminates a crystalline sample. Indexing a Laue diffraction pattern allows the crystallographic orientation, and in certain cases lattice elastic strains and defects of the sample, to be determined (Sheremetyev *et al.*, 1991 ▸; Marín & Diéguez, 1999 ▸; Maaß *et al.*, 2006 ▸; Xu *et al.*, 2017 ▸; Deillon *et al.*, 2019 ▸). With the development of Laue microdiffraction, which utilizes a focused beam, nondestructive 3D characterization can be achieved with submicrometre spatial resolution (Larson *et al.*, 2002 ▸; Larson & Levine, 2013 ▸; Hofmann *et al.*, 2012 ▸; Cornelius & Thomas, 2018 ▸; Örs *et al.*, 2018 ▸; Altinkurt *et al.*, 2018 ▸). As a consequence, Laue microdiffraction is today a major tool for materials characterization. Examples of established techniques include methods based on both synchrotron and laboratory source X-rays (Larson *et al.*, 2002 ▸; Tamura *et al.*, 2003 ▸; Levine *et al.*, 2006 ▸; Lynch, Stevenson *et al.*, 2007 ▸; Lynch, Tamura *et al.*, 2007 ▸; Hofmann *et al.*, 2012 ▸; Larson & Levine, 2013 ▸; Zhou *et al.*, 2016 ▸; Cornelius & Thomas, 2018 ▸; Örs *et al.*, 2018 ▸; Altinkurt *et al.*, 2018 ▸).

Several approaches have been developed to index Laue diffraction patterns which contain multiple spots. Traditional methods determine crystallographic orientations by matching the angles between triplets of experimental diffraction vectors and the theoretical ones (of an unstrained crystal) (Ohba *et al.*, 1981 ▸; Chung & Ice, 1999 ▸; Tamura *et al.*, 2003 ▸). Recently, a routine to preselect detected spots has been proposed (Dejoie & Tamura, 2020 ▸) to increase the robustness of the method for indexing ‘small’-unit-cell samples (which can produce a ‘large’ number of diffraction spots). To increase the reliability of these methods, a generalized Hough transform strategy has also been proposed (Morawiec & Bieda, 2005 ▸). By converting any possible matched sets (*e.g.* pairs or triplets) into points in rotation space (Morawiec, 2020 ▸), or individual spots to lines (Gevorkov *et al.*, 2020 ▸), those that match or intersect in the crystallographic orientation domain will accumulate and thus indexing robustness will increase.

With the advancement of computing power, new indexing methods based on forward simulations have recently been developed. This type of method generates a large number of Laue diffraction patterns based on dictionary crystallographic orientations and then compares them with the experimental one(s) using a correspondence measure (Gupta & Agnew, 2009 ▸). The main advantage of this approach is its robustness against low-quality Laue diffraction patterns.

A key element for these dictionary-based approaches is uniform sampling of the crystallographic orientation space (Singh & De Graef, 2016 ▸; Larsen & Schmidt, 2017 ▸; Quey *et al.*, 2018 ▸). The dictionary crystallographic orientations must match the targeted angular accuracy. In practice this leads to a huge number of dictionary patterns, even when this number is reduced by considering crystal symmetries. All the dictionary patterns have to be checked against the experimental ones, which means that this type of method is typically slow compared with those using direct triplet matching (Singh & De Graef, 2016 ▸).

In the present work, a novel route, called dictionary-based branch and bound (DBB), is proposed to mitigate this issue. This method is inspired by the branch-and-bound approach (Yang *et al.*, 2016 ▸) used to determine rigid-body rotation between two point sets in computer vision. An appropriate upper-bound criterion allows us to reach the finest angular accuracy possible while using a coarser dictionary. Thus, calculation time and memory requirements are reduced.

Another important motivation for developing DBB is to enable indexing of superimposed diffraction patterns for many crystals, which is very challenging using the triplet-matching methods. In this paper it is demonstrated that DBB can readily handle diffraction patterns from 100 crystals. Simulated data are considered for the testing approach and for incorporating typical experimental challenges: (i) spot overlap when the number of illuminated crystals is large; (ii) fake spots added to represent physical artefacts on the detector and/or oversensitive spot detection; (iii) true spots removed to simulate true spots that are undetected due to insufficient brightness compared with the noise; (iv) Poisson noise added on the detector image. Finally, as a demonstration, DBB is also used to process experimental synchrotron microdiffraction data.

## 2. Dictionary–branch–bound indexing

### 2.1. Overall route

**Figure 1:**
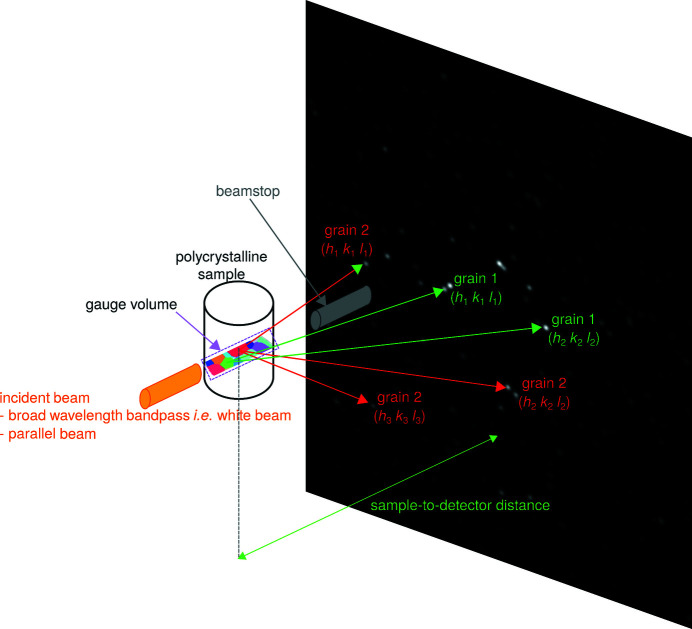
The typical diffraction setup for which DBB is applicable. In this case, the detector is placed in a transmission configuration. Arrows represent diffracted X-ray beams.

A typical (micro-)diffraction setup for which DBB is applicable is shown in Fig. 1 ▸. The broad-bandpass parallel X-ray beam is incident on a polycrystalline sample and diffracted onto the detector. The latter can be placed in any position, including transmission (as in Fig. 1 ▸), 90° reflection and back-reflection configurations.

**Figure 2:**
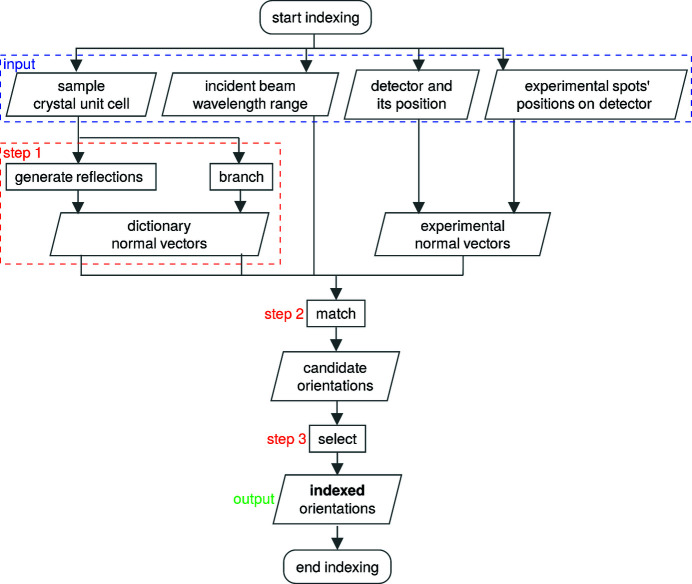
A flowchart of the dictionary–branch–bound method.

The overall route of DBB is summarized in Fig. 2 ▸. It is assumed that the crystal structure of the sample, the X-ray energy range of the broad-bandpass incident beam and the detector geometry (position and pixel information) are known. The incident beam is considered to be parallel. The distances between the centre of mass of each crystal in the sample and the centre of the gauge volume are negligible compared with the sample-to-detector distance. Finally, the positions of detected diffraction spots are used to calculate experimental unitary diffraction vectors (as the diffracted photon wavelength is unknown).

DBB consists of three main steps:

(i) The crystallographic orientation space is subdivided into chunks called branches whose coverage of the crystallographic orientation space typically corresponds to a 2–4° mis­orien­tation angle. For each branch, reflections which are expected for the branch (*i.e.* would produce a spot on the detector) are determined (Section 2.2, step 1 in Fig. 2 ▸).

(ii) For each branch, its expected reflections are matched with detected spots to construct candidate crystallographic orientations (Section 2.3, step 2 in Fig. 2 ▸).

(iii) Best-candidate crystallographic orientations are selected iteratively to index as precisely as possible the (so far) non-indexed detected spots, and then refined to constitute the indexed crystallographic orientations (Section 2.4, step 3 in Fig. 2 ▸).

### 2.2. Branch (step 1)

The fundamental region (considering crystal symmetry) of the crystallographic orientation space is sampled by a dictionary, and for each dictionary crystallographic orientation, a domain called a branch around it is defined such that the union of branches contains the fundamental region of the crystallographic orientation space. Dictionary crystallographic orientations and their branches may be outside the fundamental region of the crystallographic orientation space and/or branches may intersect, *i.e.* overlap each other, without creating any issue.

**Figure 3:**
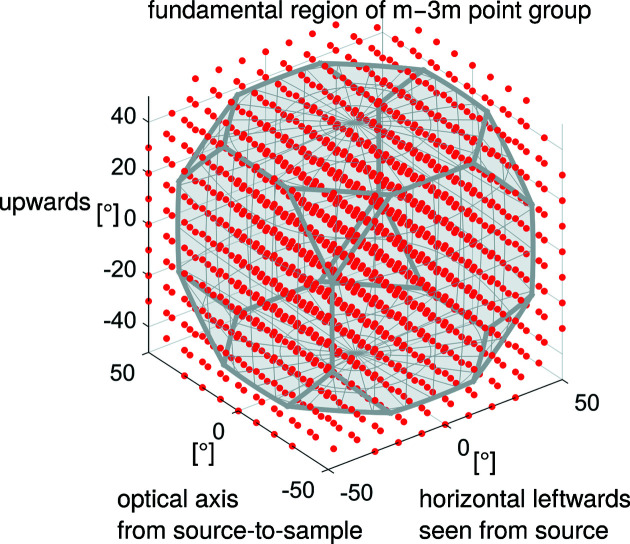
An example of a dictionary of 1300 crystallographic orientations (red dots) defined as a regular three-dimensional grid with 10° spacing in the axis–angle representation of rotations whose basis is defined from macroscopic laboratory directions. The branches (not shown for readability) are cubes centred on the dictionary crystallographic orientation 10° edges along the macroscopic laboratory directions. Some dictionary crystallographic orientations and branches are outside the fundamental region of the 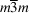 point group (grey domain). This is to ensure that they cover the whole fundamental region of the 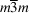 point group.

An example of a crystallographic orientation dictionary and its branches is shown in Fig. 3 ▸, where the dictionary is a regular three-dimensional grid with 10° spacing, and the branches are cubes of 10° edge length centred on the dictionary crystallographic orientations along the macroscopic laboratory directions.

For each branch, reflections which are expected to produce spots on the detector for the given setup and incident photon wavelength range are determined (Appendix *A* [App appa]); these are called expected reflections (ERs). Reflections leading to identical unitary normal vectors are merged into one, though the photon wavelength for Bragg diffraction of each reflection is considered separately. Identical unitary normal vectors of ERs for the dictionary crystallographic orientation of the branch are called expected dictionary normal vectors (EDNVs) and will be used for matching (Section 2.3).

### 2.3. Match (step 2)

In the match step, the following process is repeated for each branch. First, each EDNV of the *N* + N* strongest ERs for the branch is compared with each of the experimental normal vectors to find potential matches (Section 2.3.1) under a specific criterion. *N* is the number of ERs for the branch which will be used to construct candidate crystallographic orientations. However, instead of testing only *N* ERs for the branch, it is possible to test N* extra ones to render the match more resilient to undetected true spots. Secondly, all possible candidate combinations are constructed using *N* EDNVs for the branch and their *N* sets of possible matches among the *N* + N* tested ERs for the branch. Associated candidate crystallographic orientations are calculated (Section 2.3.2).

#### 2.3.1. Matching criterion

**Figure 4:**
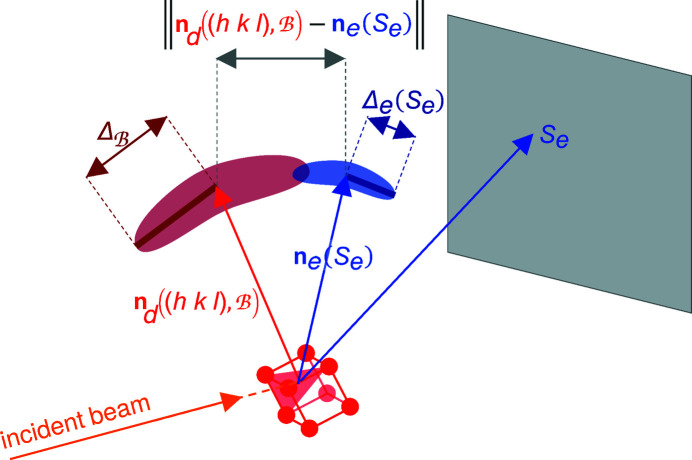
A sketch showing the matching criterion between the expected dictionary normal vector {red arrow, 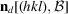 } of an ER (*h* *k* *l*) in the branch 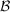 , and the normal vector [blue vector, **n**_*e*_(*S*_*e*_)] of a detected spot *S*_*e*_. The EDNV 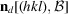 belongs to the red semi-transparent domain delimited by the branch looseness distance 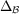 . The true (unknown) normal vector belongs to the dark-blue semi-transparent domain delimited by the uncertainty distance Δ_*e*_(*S*_*e*_) on the experimental normal vector **n**_*e*_(*S*_*e*_) of the detected spot *S*_*e*_. In the figure 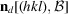 is a possible match for **n**_*e*_(*S*_*e*_) in 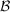 .

For each of the *N* + N* strongest ERs of a branch, the EDNV 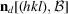 is compared with the experimental normal vector **n**_*e*_(*S*_*e*_) of each detected spot *S*_*e*_ to check for a possible match using the following criterion: 

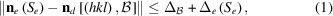

where the upper bound on the right-hand side of equation (1) is composed of the branch looseness distance 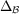 and the uncertainty distance Δ_*e*_(*S*_*e*_) on the experimental normal vector **n**_*e*_(*S*_*e*_) of the detected spot *S*_*e*_ (Fig. 4 ▸).

The branch looseness distance 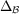 covers the difference between the dictionary crystallographic orientation and any crystallographic orientation in the branch. More rigorously, 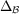 is an upper bound for the norm of the variation of a unit vector when rotating from the dictionary crystallographic orientation to anywhere in the branch. The branch looseness distance 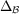 is calculated as

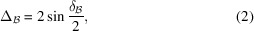

where the branch looseness angle 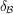 is an upper bound for the angular deviation of any vector when rotating from the dictionary crystallographic orientation to anywhere in the branch. Hence 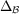 depends on how the dictionary and branches are constructed, and can be derived by geometric considerations.

The uncertainty distance Δ_*e*_(*S*_*e*_) on the experimental normal vector **n**_*e*_(*S*_*e*_) of the detected spot *S*_*e*_ covers the difference between the detected spot and the (unknown) true spot. More precisely, for a detected spot, it is an upper bound of the norm of the difference between the normal vectors associated with the detected spot and the true spot. This uncertainty distance Δ_*e*_(*S*_*e*_) can come from (i) the uncertainty in detecting the spot position on the detector, which could originate from elastic/plastic strain and/or noise in the detector signal, (ii) the fact that the crystal has a physical size within the sample which deviates from the hypothesis that all diffracted beams come from its centre, and (iii) the divergence of the non-perfectly parallel beam. The calculation of Δ_*e*_(*S*_*e*_) is presented in Appendix *B* [App appb].

Here, distances are calculated as the norm of differences (*i.e.* informally the distances between arrow tips) between (unitary) vectors rather than angles. In this way, the triangular inequality guarantees that the sum of 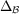 and Δ_*e*_(*S*_*e*_) is an adequate upper bound.

#### 2.3.2. Construct candidate crystallographic orientations

**Figure 5:**
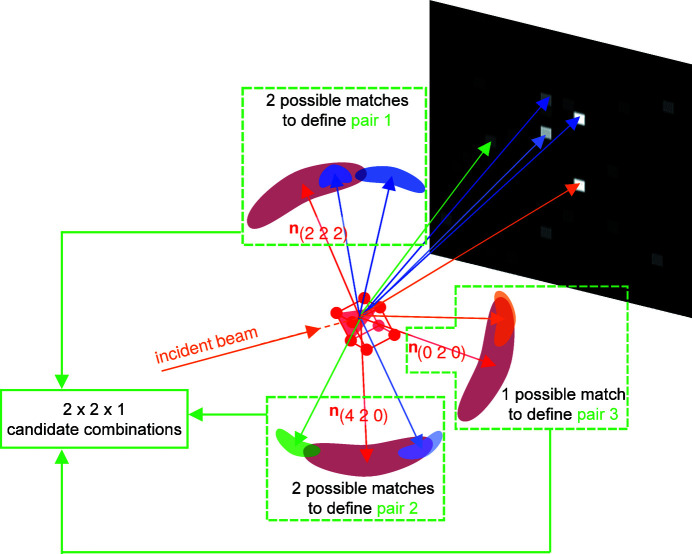
A sketch showing the construction of candidate combinations from possible matches between EDNVs and experimental normal vectors. In the figure, *N* = 3, the three red arrows are the EDNVs of the three strongest ERs in the branch and other coloured vectors are experimental normal vectors. In total 2 × 2 × 1 = 4 candidate combinations can be constructed for this branch.

For each one of the *N* + N* tested EDNVs in the branch, there can be several possible matches among the experimental normal vectors (Fig. 5 ▸). Candidate crystallographic orientations are constructed by aligning *N* EDNVs out of the *N* + N* tested ones and their possible matches among the experimental normal vectors.

Let us first define *N*_pm_ (≤ *N* + N*) as the number of EDNVs (out of the *N* + N* tested ones) with at least one possible match among the experimental normal vectors. If *N*_pm_ < *N*, the number *N*_pm_ is insufficient to construct candidate combinations, so this branch is not considered further. If *N*_pm_ ≥ *N*, then all choices of *N* EDNVs among these *N*_pm_ which each have at least one possible match^1^ are considered to construct all possible candidate combinations (Fig. 5 ▸).

For each candidate combination, the associated candidate crystallographic orientation is calculated by aligning as precisely as possible the *N* EDNVs to their corresponding *N* experimental normal vectors. This is done by minimizing the least-squared norms of the differences between vectors (*i.e.* solving Wahba’s problem; Wahba, 1965 ▸) weighted by the squared inverse of distance uncertainties through a singular value decomposition (Markley, 1988 ▸) [similar to the procedure used by Gupta & Agnew (2009 ▸)].

### 2.4. Select (step 3)

The best crystallographic orientations are selected among the candidate ones and then refined, which results in the final indexed crystallographic orientations.

The selection is done iteratively in three steps: (i) identify the best remaining (*i.e.* non-selected) candidate crystallographic orientation, (ii) check if it satisfies two criteria (detailed below) and (iii) if it does, select it, *i.e.* append it to the set of indexed crystallographic orientations. This process stops when the best remaining candidate does not satisfy the criteria, at which point DBB indexing also stops.

Intuitively, the best remaining candidate is the one which best matches the so far non-indexed detected spots. To implement this in practice, for any input set of crystallographic orientations, a score *s* is associated with each detected spot *S*_*e*_, defined as

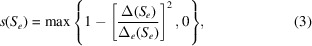

where Δ(*S*_*e*_) is the norm of the difference between the experimental normal vector of the detected spot *S*_*e*_ and the closest of the unitary normal vectors of all reflections (not only the ones of the *N* + N* strongest ERs) for all crystallographic orientations in the input set. The score of a detected spot *S*_*e*_, *s*(*S*_*e*_), quantifies how well it is indexed by the input set of crystallographic orientations; a detected spot is considered as indexed if, and only if, its score is positive.

The best remaining candidate is then identified as follows. The selected crystallographic orientations are considered as the input set, and the detected spots which are not indexed (*i.e.* whose score is zero) are determined and referred to as ‘currently not indexed’. Each remaining candidate is then appended to the selected crystallographic orientations to form the input set and the score of each currently not indexed detected spot is calculated, before summation. The best remaining candidate is the one which maximizes this sum, hereafter denoted Δ*S*. The number Δ*n* of detected spots newly indexed by the best remaining candidate is also calculated.

The best remaining candidate is selected if, and only if, it satisfies both of the following criteria:

(i) Δ*S* is greater than a fraction *f*_thr_ of the mean of the Δ*S* values associated with previously selected crystallographic orientations. The fraction *f*_thr_ has been chosen empirically to equal 1/4 as the best value, using simulations of X-ray Laue diffraction.

(ii) Δ*n* is greater than a user-defined value Δ*n*_thr_.

Finally, each selected crystallographic orientation is refined, taking diffraction spots associated with all ERs into account (using the same method as for the creation of candidate crystallographic orientations). Refined selected crystallographic orientations are the indexed ones.

DBB has been implemented in MATLAB and uses the open source *MTEX* toolbox (https://mtex-toolbox.github.io/) for calculations involving rotations, crystallographic orientations and crystal symmetries. The code is available on request.

## 3. Performance evaluation of DBB using simulated data

To illustrate the performance of DBB and understand the influence of its settings, as well as to provide practical guidelines for their choice, DBB has been tested using simulated diffraction data.

Several types of artefacts which may be caused by typical experimental issues are considered, including (i) the point spread function, (ii) fake spots due to detector noise, (iii) undetected true spots due to low spot intensity and (iv) background noise. In this way, the most important issues that DBB will face when indexing real experimental data are considered. The advantages of this simulation-based approach over experiments include (i) the ground truth is known and (ii) the effects of each experimental issue are separated from other issues, which eases understanding.

### 3.1. Method

The diffraction setup shown in Fig. 1 ▸ was considered for the simulations. The geometric ray-tracing approach was used to simulate the path of the X-rays. Even though only the associated normal vectors are used as input for DBB, the intensities of the spots were also calculated because intensity may affect spot detection. The sample was simulated as a set of aluminium crystals, where (i) each crystal was a material point, *i.e.* had no volume, (ii) all crystals were superimposed in the same sample position and (iii) all crystals had a different crystallographic orientation. This allowed us to model all diffracted X-rays as emerging from one unique point in space.

**Figure 6:**
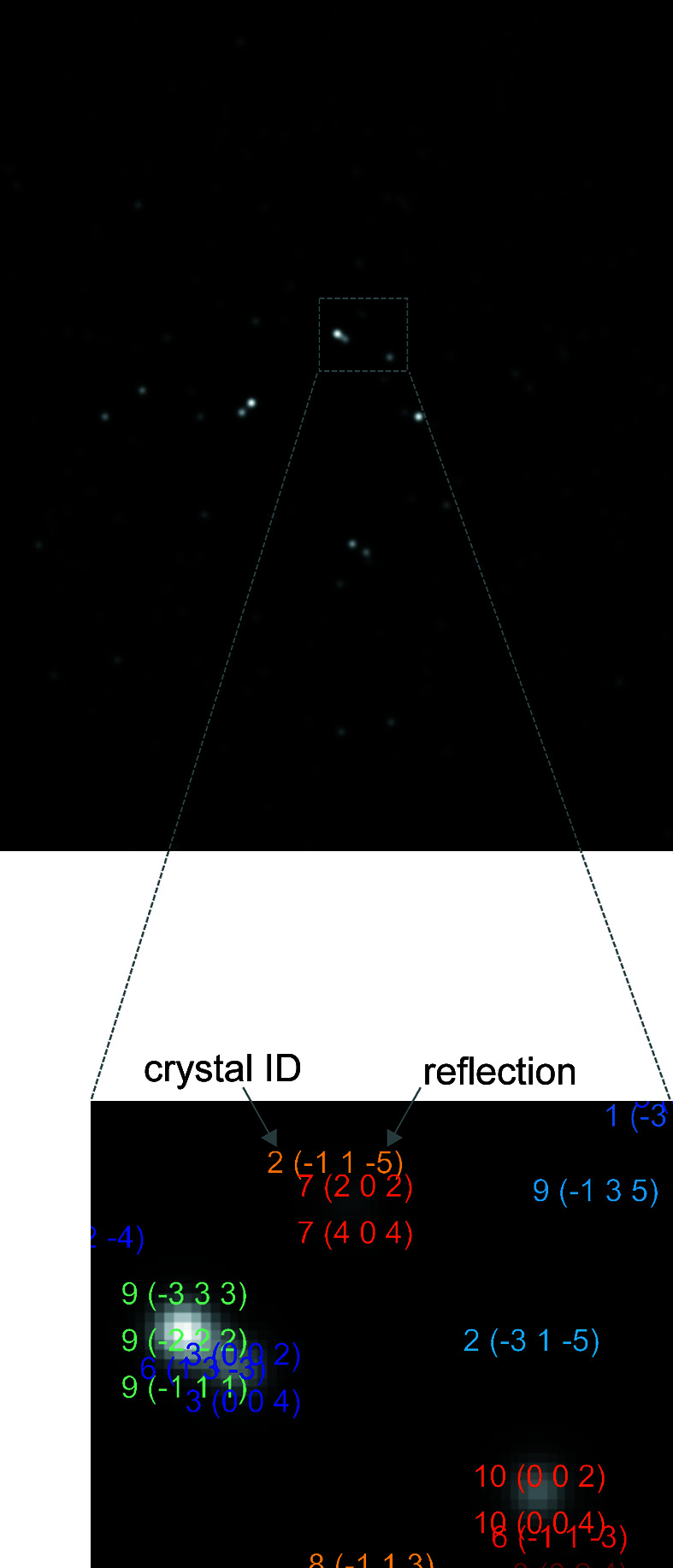
A detector image simulated by a geometric ray-tracing approach. Case 1 (ten crystals – see Table 1 ▸) is considered here. The magnified inset shows a label displayed for each spot by the program, identifying the associated crystal (by a number) and the reflection(s).

A Gaussian convolution was applied to simulate the point spread function of a typical detector and thus to mimic the typical experimental signal of a spot in an experiment (for details, see Section S1 in the supporting information). An example of a simulated detector image is shown in Fig. 6 ▸.

Three samples with ten, 50 and 100 crystals of random crystallographic orientations were considered first to study the influence of spot overlap. To mimic other experimental challenges, the detector image from the 100-crystal sample was further processed by (i) adding fake spots (10% of the real ones), each with an intensity equal to the minimum true spot intensity, (ii) randomly removing 25% of the true spots and (iii) adding Poisson noise (using the MATLAB imnoise function), such that for each pixel the expected value of the Poisson distribution was equal to the pixel signal value before applying the Poisson noise. Hence, the noise level was comparable to the true signal level.

Spots were detected by an automatic method based on normalized cross correlation with a Gaussian template, where local maxima above a certain threshold *t* in the interval [0; 1] were kept as detected spots. For noise-free images, *t* = 0.05 was used, whereas for noisy images higher thresholds *t* were chosen (see Table 1 ▸) to eliminate bad spots coming only from the noise.

For the indexing, the dictionary and branches were constructed like in the example shown in Fig. 3 ▸ with a dictionary resolution θ_dict_ of 4°. This construction of the dictionary and branches allowed us to determine a valid 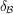 as a function of the dictionary resolution θ_dict_ [obtained from lemma 1, *i.e.* inequations (6), in the work of Yang *et al.* (2016 ▸)], 

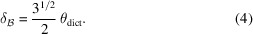

**Figure 7:**
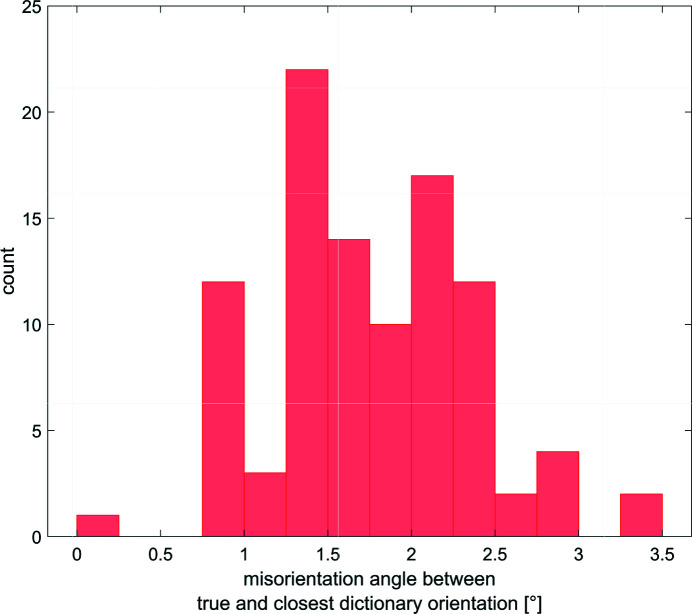
The distribution of the misorientation angles for each of the 100 ground-truth crystallographic orientations common to cases 3–15 (see Table 1 ▸) to the closest dictionary crystallographic orientation.

With this dictionary, the maximum misorientation angle between any of the 100 crystallographic orientations and the closest dictionary crystallographic orientation was 3.43° (distribution in Fig. 7 ▸), which is below the upper bound of 3.46° determined by equation (4).

The distribution (Fig. 7 ▸) shows that 99% of the ground-truth crystallographic orientations are misoriented by more than 0.5° from the dictionary ones. This constituted a critical test for DBB, which had to retrieve the ground-truth crystallographic orientations starting from a dictionary which was off by more than 0.5° and up to 3.43° in terms of misorientation angle.

In total, 15 test cases were studied (Table 1 ▸). To quantify the indexing quality for the test cases, several parameters were defined, as follows.

(i) The angular uncertainty δ_*e*_(*S*_*e*_) on the experimental normal vector for a detected spot *S*_*e*_ was determined on the basis of (*a*) the 3/2 detector pixel diagonal used as the uncertainty Δ_*d*_ on detected spot position on the detector and (*b*) the detector setup (Appendix *B*2). Note that δ_*e*_(*S*_*e*_) depends on the spot position on the detector, and is larger for spots close to the centre of the transmitted beam.

(ii) The mean angular uncertainty on experimental normal vectors 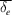 was calculated as the mean over all detected spots. Thus 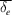 represents the typical angular uncertainty one may expect for the setup and was used to evaluate the indexing quality. A ground-truth crystallographic orientation is correctly indexed if, and only if, there is an indexed crystallographic orientation closer than 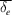 in terms of misorientation angle. A false negative (FN) is a ground-truth crystallographic orientation which is not correctly indexed. A false positive (FP) is an indexed crystallographic orientation for which there is no ground-truth crystallographic orientation closer than 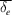 in terms of misorientation angle.

(iii) The mean angular error 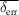 is defined as the mean, over all correctly indexed ground-truth crystallographic orientations, of the misorientation angle of each one with its closest indexed crystallographic orientation. It was determined to evaluate the angular accuracy of the indexed crystallographic orientations. Thus 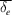 indicates an upper bound for an acceptable 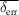 between input and indexed crystallographic orientations.

The indexing setting *N* was set to 3, N* to different values (0, 1 and 2) (see Table 1 ▸) and Δ*n*_thr_ to 4.

All computations (simulation of the detector images and DBB indexing) were performed on a Hewlett Packard Prodesk 600G5 Small Form Factor personal computer equipped with an Intel Core i9 9700 central processing unit and 64 GB of random access memory.

### 3.2. Results and discussion

The detected spots differed between cases (details of the spot detection are presented in Section S2 in the supporting information). High numbers of detected spots tended to reduce the differences in 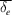 between the different cases (Table 1 ▸). For each case, the mean angular uncertainty on experimental normal vectors was 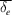 = 0.60°. Among all detected spots over all cases, the lowest and highest δ_*e*_(*S*_*e*_) were 0.17 and 3.40°, respectively.

When the number of crystals in the sample increases, the average number of detected spots per crystal decreases as a result of spot overlap. As shown in Table 1 ▸, for case 3 only 1872 spots out of the 2715 true ones (*i.e.* 69%) were detected. This induced a larger spot shift (Fig. S1 in the supporting information).

**Figure 8:**
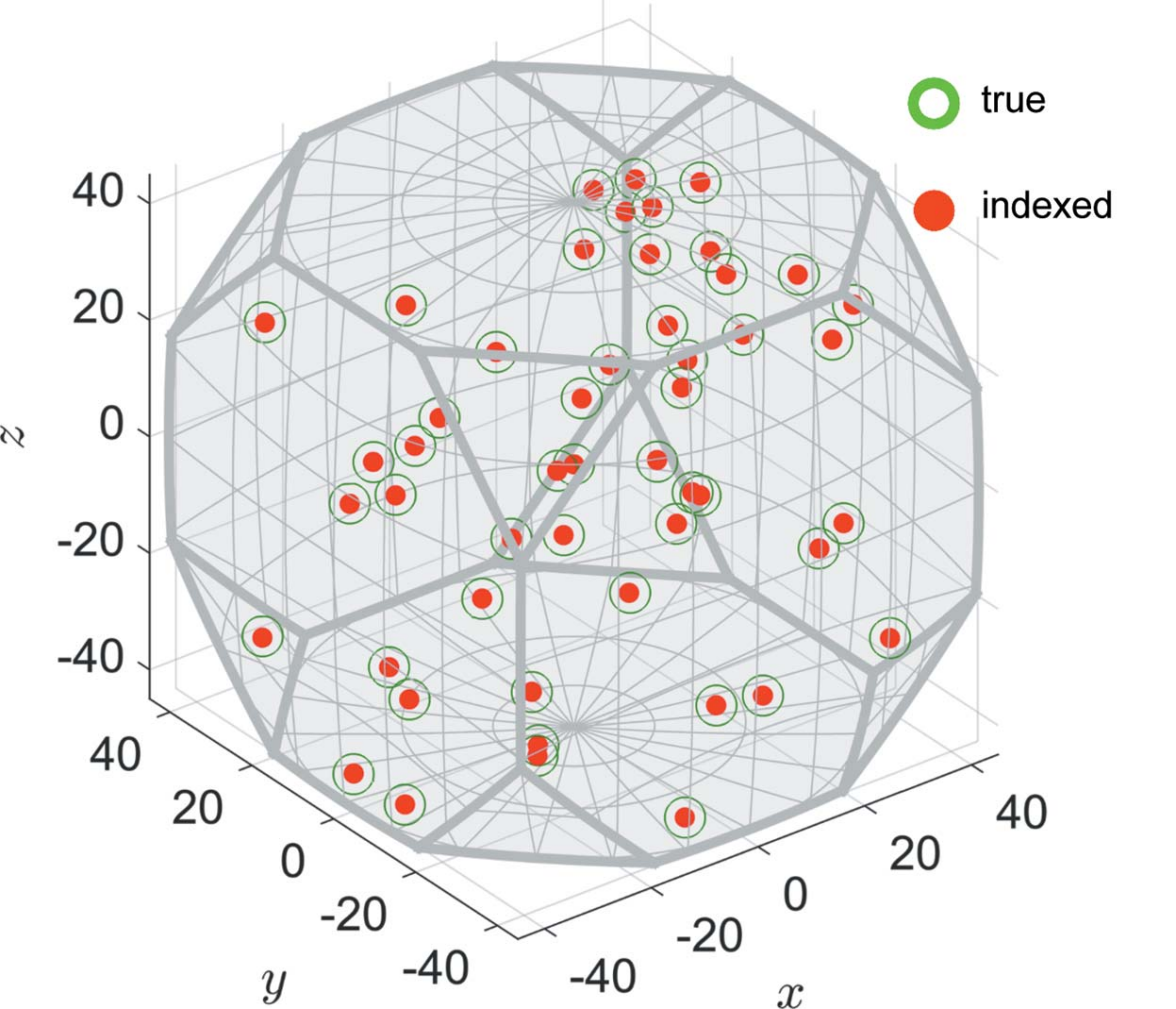
An axis–angle representation of the input (green rings) and indexed (red circles) crystallographic orientations for case 2 (50 crystals). The grey domain is the fundamental region of the point group 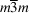 of the aluminium crystal structure.

For cases 1 and 2 with less severe spot overlap compared with case 3, DBB indexing is fully satisfactory (Fig. 8 ▸): no FNs or FPs are deduced, but the mean angular error 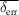 increases from case 1 to case 2 because of more spot overlap. For case 3 (with 100 crystals), DBB indexing resulted in two FNs and two FPs. This is because spot overlap led to inaccurate detected spot positions on the detector, *i.e.* the detected spot positions were further away from the true spot positions than the chosen Δ_*d*_ value of the 3/2 detector pixel diagonal. Thus some detected spots were not captured as possible matches for the *N* ERs, and the two associated correct candidates were not created, leading to the two FNs. They were replaced by two other candidates to index the ‘orphan’ (which should have been indexed by the two FNs) detected spots, leading to two FPs.

The problem could be overcome by increasing the DBB setting N* from 0 (case 3) to 1 (case 4), where a fully satisfactory indexing of the 100 ground-truth crystallographic orientations was obtained, though with a four times longer calculation (see Table 1 ▸). The mean angular error 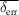 decreases as N* increases. This is because the correct matches are obtained when N* is increased.

When 296 fake spots were added (cases 5 and 6), the total number of detected spots increased by only 74 (compared with cases 3 and 4), which did not overlap with any true spots. Compared with cases 3 and 4, the results from cases 5 and 6 show that the indexing quality was not affected by the fake spots.

After random removal of one-quarter of the true spots (cases 7–9), some of them corresponding to the *N* + N* strongest ERs were eliminated and thus not captured as possible matches. Therefore, the associated correct candidates corresponding to ground-truth crystallographic orientations were not constructed, leading directly to FNs. This problem could be remedied by increasing N*. With N* = 2 (case 9) only one FP and one FN remained. Setting N* = 3 led to excessive memory requirements. However, it is believed that all the ground-truth crystallographic orientations can be correctly indexed by increasing N*, since there were on average still more than 15 spots per crystal.

The mean angular errors 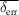 in cases 7 and 8 were higher than in cases 3 and 4, respectively. This is because fewer candidates were created due to the removal of true spots, which eliminated some which were indexed in cases 3 and 4 and were closer to the ground-truth crystallographic orientations.

For cases with Poisson noise added (cases 10–15), the expected value of the noise for each pixel was proportional to the level of the true signal of the pixel, and hence the signal was only locally degraded around the true spots (Fig. S1 in the supporting information). Even when setting the spot detection threshold *t* as high as 0.2, the detected spots were more numerous in the presence of noise (2302, case 10) than in the absence of noise (1872, case 3). This implies that for some detected spots in case 3 without noise (each one being necessarily on a true spot) more than one neighbouring shifted detected spot was present in case 10 with noise (red arrows in Fig. S1). Detecting more neighbouring shifted spots near a true spot can perturb the indexing when calculating scores. Indeed, if for different true spots different numbers of (noise-induced) neighbouring shifted detected spots exist, they will contribute unevenly to the score. This can degrade the overall evaluation of candidates, their selection and the indexing result.

This situation could be remedied by increasing the threshold *t* and increasing N*. Increasing the threshold *t* eliminated spots produced only by the noise (red arrows in Fig. S1), which thus eliminated incorrect possible matches and FPs, as well as FNs by selecting correct candidates (cases 10, 12 and 14 in Table 1 ▸). Increasing N* helped to capture possible correct matches despite excessive spot shifts due to noise and thus helped to construct correct candidates. A perfect indexing was reached with *t* = 0.4 and N* = 1 within a reasonable time frame (case 15). Note that the number of detected spots in case 15 was only 1462, which is even fewer than for cases 7–9. This is because increasing the threshold *t* actually eliminated both poorly shaped fake spots induced by noise and faint true spots from the weakest reflections. Losing the latter ones was not problematic to DBB because they came from the weakest reflections and thus were less likely to be tested when looking for possible matches and hence of less impact for the indexing. Nevertheless, noise increased the spot shift and thus led to a higher mean angular error 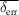 for case 15 than for case 4.

The results of these test cases show that the resilience of DBB indexing may be further improved by three approaches: (i) supplying more accurately detected spots as inputs to DBB (this is related to the spot detection rather than to DBB itself), (ii) increasing the chosen uncertainty Δ_*d*_ on the detected spot position on the detector to better handle higher shifts of detected spots (due to true spot overlap and/or noise) and (iii) increasing the value of N*, which is the most polyvalent approach, as it helps to handle both the shift of detected spots (due to true spot overlap and/or noise) and undetected true spots (due to noise).

The results show that both the number of detected spots and the setting N* affect the computational time. Theoretically, this time and the memory requirements are dominated by and proportional to the number of candidate crystallographic orientations constructed, which itself can be estimated as proportional to the time factor *f* as a function of the number of detected spots *n* (supplied as input to DBB) and the settings *N* and N*, 

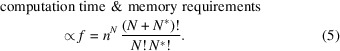

An explanation of equation (5) is detailed in Section S3 in the supporting information.

**Figure 9:**
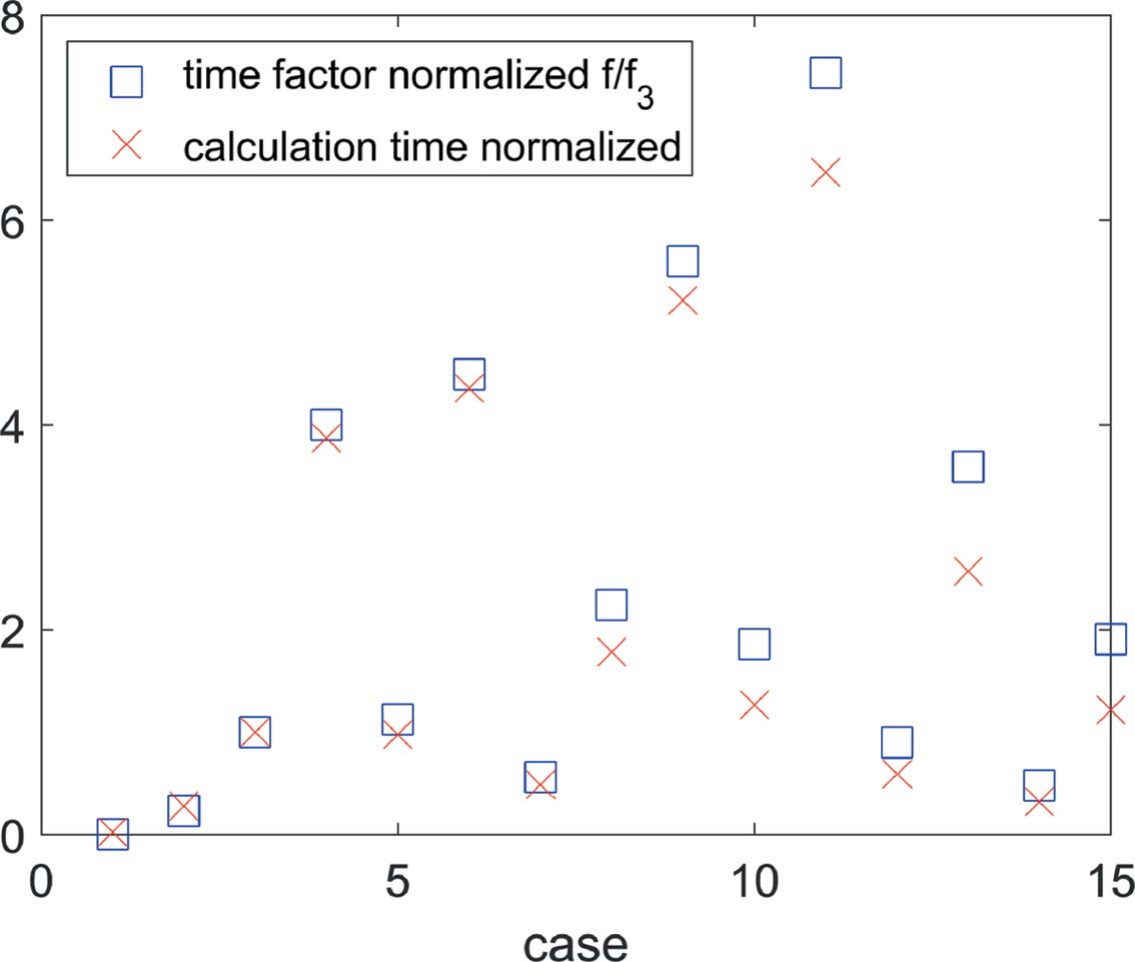
The normalized time factor *f*/*f*_3_ (red crosses) [theoretical, calculated from equation (5)] and the normalized calculation time (blue squares, experimental) for cases 1–15. Normalized refers to division by the value for case 3.

In practice, by considering a normalized time factor *f*/*f*_3_, *i.e.* the time factor divided by the time factor of case 3 (as reference), Fig. 9 ▸ shows that equation (5) predicts well the (relative) computational time for all cases. This enables the calculation of time frames for different data sets (changing the number of spots *n*) and/or settings (changing *N* and/or N*).

## 4. Application of dictionary–branch–bound to experimental data

To demonstrate further the power of DBB, it is applied to synchrotron Laue microdiffraction data. To evaluate the indexing results, depth-resolved patterns were obtained using a differential aperture and indexed using the *LaueGo* software package (https://www-stg.aps.anl.gov/Science/Scientific-Software/LaueGo).

### 4.1. Methods

Synchrotron Laue microdiffraction data were acquired on beamline 34-ID-E at the Advanced Photon Source, Argonne National Laboratory, USA (Larson *et al.*, 2002 ▸; Yang *et al.*, 2004 ▸). The incident beam was a parallel beam with a Lorentzian profile and a full width at half-maximum of 300 nm defined by a set of non-dispersive Kirkpatrick–Baez mirrors. The sample was spark-plasma-sintered aluminium with a mean crystal size of 5 µm (Zhang *et al.*, 2020 ▸). It was mounted at an inclination angle of 45° to the incident beam. A Perkin–Elmer square flat-panel detector was placed horizontally 511 mm above the sample (hence in a 90° reflection configuration) with one edge along the incident beam. The detector presents 2048 × 2048 pixels and a pixel edge length of 200 µm. One randomly chosen position of the sample was considered for the present testing. A platinum knife-edge wire was used as a differential aperture for resolving depth information. The aperture was scanned in a plane parallel to the sample surface at a distance of 250 µm, on which basis 193 individual depth-resolved detector images from the sample surface to a depth of 193 µm below the sample surface along the incident beam were obtained.

These 193 depth-resolved detector images were each indexed using the *LaueGo* software package, associating each image with the best possible indexed crystallographic orientation (though the indexing can lead to several indexed crystallographic orientations), leading to 193 *LaueGo* crystallographic orientations. Duplicates were removed using a misorientation angle threshold of 0.2°, leading to 53 *LaueGo* crystallographic orientations.

The 193 depth-resolved detector images were then summed into a single detector image (termed ‘merged’) which was indexed by DBB, leading to what are hereafter called DBB crystallographic orientations.

The spots were detected from the merged detector image using the same method as for the simulated data (Section 3.1), selecting local maxima of normalized cross correlation between the detector image and a Gaussian template which were greater than the chosen threshold *t* = 0.32.

For DBB indexing, the following settings were used: θ_dict_ = 1°, Δ*n*_thr_ = 4, Δ_*d*_ = 1.5 pixel edge lengths and *N* = 3, and values from 0 to 3 were tested for N*. In this configuration, a dictionary resolution θ_dict_ of 4° led to capturing too many possible matches and the construction of too many candidates, resulting in a lack of memory to store them. Note that, compared with the simulated case (Section 3), the larger sample-to-detector distance refines the achievable angular accuracy, quantified by the mean angular uncertainty on experimental normal vectors 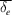 of 0.0049° for the detected spots.

Finally, the *LaueGo* and DBB crystallographic orientations were compared using a misorientation angle threshold of 0.0049° + arctan (193 µm/511 mm) + 0.2° = 0.23°, *i.e.* if they do not deviate by more than 0.23° then they are considered identical.

### 4.2. Results

**Figure 10:**
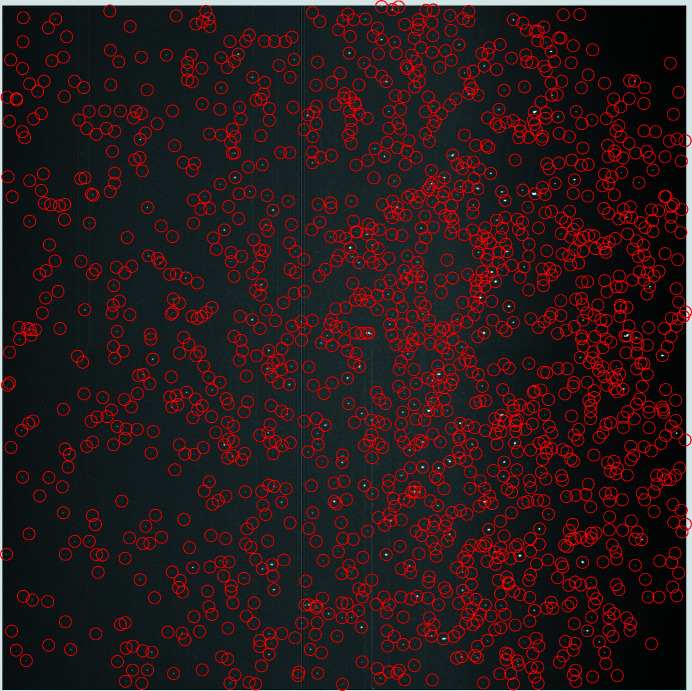
A merged detector image with the 1558 detected spots marked by red rings.

There are 1558 detected spots in the merged detector image (Fig. 10 ▸).

The DBB indexing results are presented in Table 2 ▸. If the 53 *LaueGo* crystallographic orientations can be considered as the ground truth, then the successfully indexed crystallographic orientations and their mean angular error 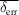 , false negatives and false positives are as reported in Table 2 ▸. As already observed for simulated data (Section 3), increasing N* leads to more successfully indexed crystallographic orientations, and the number of indexed detected spots increases accordingly. The associated mean angular error 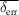 is, as expected, less than the 0.23° angle chosen for matching, but it is greater than the 0.0049° mean angular uncertainty on experimental normal vectors taking only the uncertainty on the detected spot positions into account, which suggests that both the crystal position within the sample and the heterogeneities of crystallographic orientations within one grain have an impact on the angular accuracy. For the case N* = 3, 46 out of the 53 *LaueGo*-merged crystallographic orientations are successfully indexed by DBB, *i.e.* there are seven FNs. This is considered a very promising result. However, there are 103 FPs.

The proportion of FPs is surprisingly high. This may be caused by spots becoming visible when summing the individual depth-resolved detector images into the merged detector image, which increases the signal-to-noise ratio, *i.e.* some of these ‘false’ positives are actually true crystallographic orientations successfully indexed by DBB. It may also be related to the experimental noise, like cases 10 and 11 in Section 3. Since the present *LaueGo* results do not constitute a guaranteed ground truth, more work is needed to verify the DBB indexing results. Nevertheless, it is encouraging that such a high fraction of the *LaueGo* crystallographic orientations can be indexed by DBB when only one single merged detector image is used.

## 5. Summary

A method, called dictionary–branch–bound, has been developed to determine the crystallographic orientation of multiple (at least up to 100) crystals simultaneously illuminated by a parallel broad-bandpass X-ray beam, using only the spot positions as input.

DBB has been tested on simulated data considering a typical experimental setup. Several (ten to 100) aluminium crystals with randomly selected crystallographic orientations were illuminated by a broad-bandpass X-ray beam and the detector images were simulated. Spots were detected by an in-house detection method and their positions were supplied as input to DBB. Additional cases were considered to mimic experimental difficulties: fake spots added to test the resilience of DBB against detector artefacts and/or an oversensitive spot detection, true spots randomly removed to test the resilience of DBB against undetected true spots due to spot overlap and/or background noise, and Poisson noise added to the detector image. With proper parameters, DBB can determine all the crystallographic orientations. The coarse dictionary resolution (typically 4°) allows faster calculations than the ordinary dictionary methods while maintaining the desired angular accuracy (typically 0.05°). DBB was also tested on experimental synchrotron microdiffraction data and notably indexed 46 out of the 53 crystals detected by *LaueGo* with a deviation of less than 0.04°.

The robustness of DBB comes from its combination of (i) the guarantee of a geometrically derived upper bound to detect a possible match between an expected reflection and a detected spot, (ii) the fact that spots are not eliminated on the fly if found as a possible match for a reflection, but instead are kept during the whole process of matching with reflections, (iii) the construction of all possible candidate crystallographic orientations, and (iv) the score strategy which makes DBB robust against fake spots.

Guidelines in the choice of DBB settings have also been obtained on the basis of the tests: setting *N* to 3 works well in practice, and increasing N* is a helpful and polyvalent approach both to capture more true crystallographic orientations and to improve their angular accuracy, as it helps to deal with both shifted and undetected true spots.

## 6. Related literature

For further literature related to the supporting information, see Dectris (2021 ▸), *Online Dictionary of Crystallography* (2021 ▸) and Wolfram Research (2021 ▸).

## Supplementary Material

Additional theory. DOI: 10.1107/S1600576722006021/nb5318sup1.pdf

## Figures and Tables

**Figure 11:**
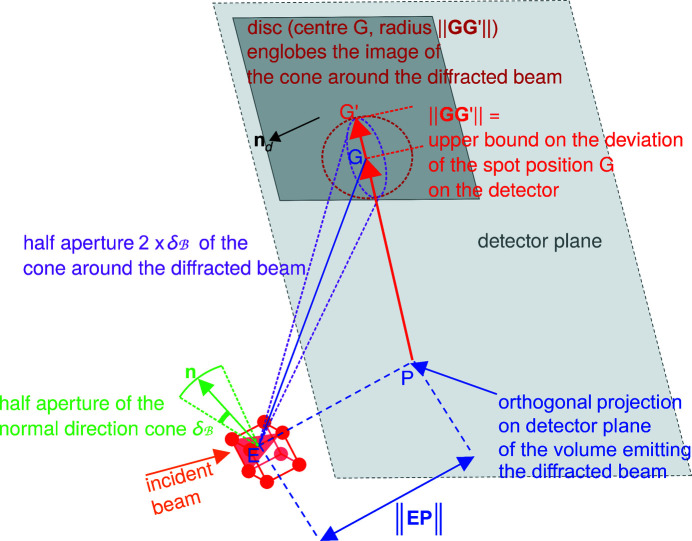
The determination of whether or not the spot position of a reflection is guaranteed to remain on the detector as the crystallographic orientation may take any value in a branch. The normal-direction angular bound 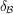 is notably converted to an upper bound ∥**GG**′∥ on the deviation of the spot position on the detector.

**Figure 12:**
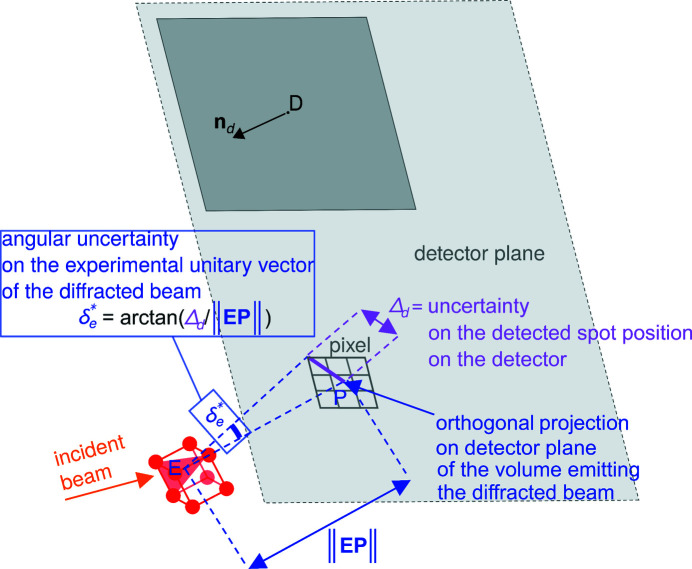
The angular uncertainty on the experimental unitary vector of the diffracted beam 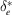 calculated by considering both (i) the distance ∥**EP**∥ from the emission point *E* in the sample to the detector and (ii) the uncertainty Δ_*d*_ in the detected spot position on the detector assuming that the true spot position is in the pixel window centred on the pixel where the detected spot position lies.

**Figure 13:**
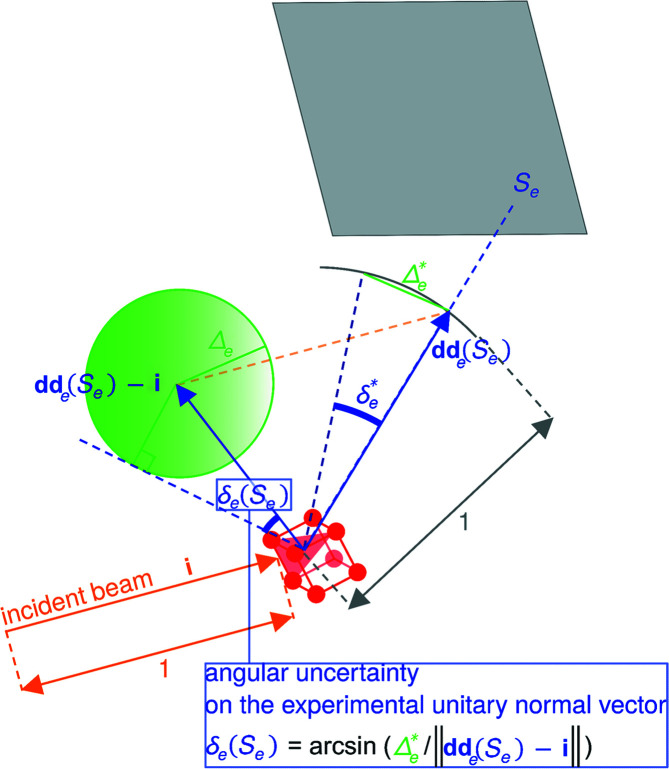
The angular uncertainty on the experimental normal vector δ_*e*_ calculated using the experimental unitary vector of the diffracted beam **dd**_*e*_(*S*_*e*_) and its angular uncertainty 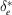 .

**Table 1:** Results of DBB indexing on simulated data run for 15 cases

Case	No. of crystals	No. of spots	No. of fake added (+) or true removed (−) spots	No. of detected spots	*t*	N*	No. of indexed crystals	No. of FNs	No. of FPs	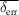 (°)	Calculation time (min)
1	10	276	0	272	0.05	0	10	0	0	0.04	14
2	50	1373	0	1145	0.05	0	50	0	0	0.06	16
3	100	2715	0	1872	0.05	0	100	2	2	0.07	57
4	100	2715	0	1872	0.05	1	100	0	0	0.05	222
5	100	2715	+296	1946	0.05	0	100	2	2	0.07	56
6	100	2715	+296	1946	0.05	1	100	0	0	0.05	249
7	100	2715	−679	1543	0.05	0	108	16	24	0.12	28
8	100	2715	−679	1543	0.05	1	99	2	1	0.07	108
9	100	2715	−679	1543	0.05	2	100	1	1	0.06	300
10	100	2715	0	2302	0.2	0	153	7	60	0.08	72
11	100	2715	0	2302	0.2	1	152	3	55	0.07	365
12	100	2715	0	1806	0.3	0	115	4	19	0.08	34
13	100	2715	0	1806	0.3	1	114	0	14	0.07	145
14	100	2715	0	1462	0.4	0	106	7	13	0.08	18
15	100	2715	0	1462	0.4	1	100	0	0	0.08	60

**Table 2:** Results of DBB indexing on depth-resolved detector image

N*	No. of indexed crystals	No. of detected spots indexed	No. of successfully indexed *LaueGo*-merged crystallographic orientations	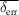 (°)	No. of FNs	No. of FPs
0	94	752	36	0.038	17	58
1	128	1017	44	0.036	9	83
2	144	1170	44	0.033	9	99
3	150	1230	46	0.032	7	103

## Footnotes

1 So there are

choices.

## Appendix A Assessing whether a reflection is expected for a branch

To check whether a reflection is expected for a branch, *i.e.* whether a crystal with a crystallographic orientation in this branch may produce a spot from this reflection on the detector, two conditions must be satisfied simultaneously for any crystallographic orientation in the branch: (i) the diffracted beam hits the detector and (ii) the photon energy for diffraction is in the range of the incident beam. This assessment is based on the dictionary resolution θ_dict_, and the geometric reasoning is detailed below.

The starting point is to consider  , which by definition bounds the angular deviation of any vector when rotating from the dictionary crystallographic orientation to anywhere in the branch. As shown in Fig. 11 ▸,  defines a ‘normal direction cone’ of half the aperture angle  (green dashed cone in Fig. 11 ▸) around the unitary normal vector of the reflection for the dictionary crystallographic orientation. It is then converted into an angular bound  on the direction of the diffracted beam (dark blue in Fig. 11 ▸), which defines a cone of half the aperture angle  (dark-magenta dashed cone in Fig. 11 ▸) around the direction of the diffracted beam.

Let us denote as *G* the position on the detector plane of the spot position for the dictionary crystallographic orientation of the branch (Fig. 11 ▸). Note that *G* is necessarily inside the detector area, otherwise the reflection would already be known as not expected for the branch. Geometrically, for a constant angular deviation  around the direction of the diffracted beam, the spot position will deviate most from *G* to another position *G*′ when the deviated direction of the diffracted beam is such that **PG** and **GG**′ are collinear and in the same direction, where *P* is the orthogonal projection on the detector plane of the centre *E* of the volume of the crystal emitting the diffracted beam in the gauge volume. The upper bound on the displacement from *G* can be calculated in practice as

where ∠(**EP**, **EG**) is the (non-oriented, *i.e.* always positive) angle between **EP** and **EG**. Then ∥**GG**′∥ is compared with the distance between *G* and the closest border of the detector to check the first condition.

The second condition is checked by verifying whether the wavelength interval for diffraction of the reflection [max{0, 2*d*sin(θ − Δθ)}; 2*d*sin(θ + Δθ)] (when the angular uncertainty  is considered) is fully included in the wavelength range of the incident beam.

Note that a coarser dictionary resolution leads to a larger upper bound on the deviation of the spot position on the detector ∥**GG**′∥ and a larger wavelength interval [max{0, 2*d*sin(θ − Δθ)}; 2*d*sin(θ + Δθ)], and thus fewer ERs for a branch. The dictionary resolution must then be chosen to be sufficiently fine to obtain enough, *i.e.* at least *N* + *N**, ERs for each branch for a given setup.

## Appendix B Uncertainty on the experimental normal vector of a detected spot

When a detected spot position deviates from its true spot position, due for example to detector background noise, or elastic or plastic strain, such a deviation will propagate to the experimental normal vector of this detected spot. Thus an upper bound Δ_*d*_ for the difference between the detected and true spot positions on the detector is chosen, and an upper bound Δ_*e*_(*S*_*e*_) on the associated norm of the variation of the experimental normal vector is derived.

### b1. Uncertainty on the experimental unitary vector of the diffracted beam

As shown in Fig. 12 ▸, the centre *E* of the volume of the crystal emitting the diffracted beam is approximated as the centre of the gauge volume, which is then projected onto the detector plane to supply the point *P*. The uncertainty (thick magenta line in Fig. 12 ▸) Δ_*d*_ on the detected spot position on the detector is considered at *P* because it is the location maximizing the angular uncertainty induced on the diffracted beam. As shown in Fig. 12 ▸, the angular uncertainty on the experimental unitary vector of the diffracted beam  is an upper bound on the angular deviation of the diffracted beam from the true one to the experimental one, which can be defined as follows:

Then the distance uncertainty on the experimental unitary vector of the diffracted beam  is an upper bound on the norm of the variation in the unitary vector of the diffracted beam from the true one to the experimental one, which can defined as follows:

### b2. Uncertainty on the experimental normal vector

The distance uncertainty  , applied initially at the arrow tip of the experimental unitary vector of the diffracted beam **dd**_*e*_(*S*_*e*_), is ‘translated’ (by −**i**, the unitary vector of the incident beam) to the tip of the experimental unnormalized normal vector **dd**_*e*_(*S*_*e*_) − **i** (Fig. 13 ▸). The distance uncertainty  then allows us to define an uncertainty ball (green circle in Fig. 13 ▸) to which ‘belongs’ the arrow tip of the experimental unnormalized normal vector **dd**_*e*_(*S*_*e*_) − **i**, enabling study of the uncertainty on the normal direction. An angular uncertainty on the normal direction is attained if (i) the true normal direction deviates as much as possible from the calculated unnormalized normal vector while (ii) still intersecting the uncertainty ball. This configuration arises if the true normal direction is tangential to the ball. The upper bound of the angular deviation of the normal direction from the true one to the experimental one, also called the angular uncertainty on the experimental normal vector and denoted δ_*e*_(*S*_*e*_), can be expressed as

The angular uncertainty on the experimental normal vector δ_*e*_(*S*_*e*_) is then translated in terms of distance. This supplies an upper bound on the norm of the difference between the true normal vector and the experimental one, which is called the distance uncertainty on the experimental normal vector, denoted Δ_*e*_(*S*_*e*_) and expressed as

which is used in the matching criterion [Section 2.3.1, equation (1)].
