# Supplementary material for: Indexing of superimposed Laue diffraction patterns using a dictionary–branch–bound approach
Source: J Appl Crystallogr. 2022 Aug 24;55(Pt 5):1085–96. doi: 10.1107/S1600576722006021 (PMC9533744; doi:10.1107/S1600576722006021)
Supplement: Supplementary file 1 [file j-55-01085-sup1.pdf]

Indexing of superimposed Laue diffraction patterns using a dictionary-branch-bound approach – supplementary material

Anthony Seret,[a]* Wenqiang Gao,[b] Dorte Juul Jensen,[a] Andy Godfrey[b] and Yubin Zhang[a]

[a] *Department of Civil and Mechanical Engineering, Technical University of Denmark, Kongens Lyngby, 2800, Denmark, and* [b] *Key Laboratory of Advanced Materials, School of Materials Science and Engineering, Tsinghua University, Beijing, 100084, PR China. E-mail: arvse@mek.dtu.dk, anthony.seret@oca.eu*

Laue diffraction, superimposed patterns, indexing, crystallographic orientations

S1. Simulation of the setup and of the Laue diffraction patterns

The incident beam is a broad-bandpass parallel focused X-ray beam with constant spectrum covering the 5 keV to 45 keV photon energy range. The lattice constant a[1] is set to 4.0495 Å (Wolfram Research, Inc., 2021).

Theoretical reflections considered to simulate the detector image (forward analysis) are the same as the ones considered in the DBB (backward analysis), for consistency and convenience of the analysis. In total 16 $\{hkl\}$ crystal plane families consisting of 141 (hkl) reflections were considered.

The intensity of the diffracted beam of a reflection was calculated as the product of the intensity of the incident beam, of the squared norm of the structure

[1] edge of the *conventional* unit cell

factor and of the polarization-Lorentz factor. No particular polarization of the X-ray incident beam was considered, so the polarization-Lorentz factor applied was $\frac{1+\cos^2(2\cdot\theta_{\mathrm{Bragg}})}{2}$ where θ_{Bragg} is the angle for Bragg diffraction. The Lorentz factor applied is $\frac{1}{\sin(2\cdot\theta_{\mathrm{Bragg}})}$ (Online Dictionary of Crystallography, 2021).

The detector modelling a Dectris Pilatus R 300K version 1 (Dectris, 2021) presents a rectangular sensitive area with a width of 83.8 mm split into 487 pixels and a length of 106.5 mm split into 619 pixels. The detector was placed in transmission mode 5 cm after the sample, orthogonal to the incident beam. The width was horizontal, the length vertical and the center on the optical axis.

The quantum efficiency of the detector sensor was also taken into account. The *detected* intensity *i.e.* photon number per time unit was calculated as the product of the diffracted beam intensity and of the detector quantum efficiency calculated from the material (silicon) and thickness (320 µm) of the sensor.

To simulate detector point spread and the calculated one pixel spots, the detector image and a Gaussian template (standard deviation of 3 pixel edge lengths) were convoluted to broaden the spots.

S2. Spot detection

Results of the spot detection method used in this work are illustrated in Figure S1. It is evident that spot overlapping can lead to shift from the true spot positions to the detected spot positions (yellow squares in figure S1a), and noise amplifies this and/or even creates fake spots (red arrows in figure S1b). Statistical analysis of spot shift for cases 3 and 10 is presented in figure S2. The proportion of spot shift larger than $\Delta_d = 3/2$ pixel diagonal used in this study is 23.7 % for case 3 and 29.7 % for case 10.

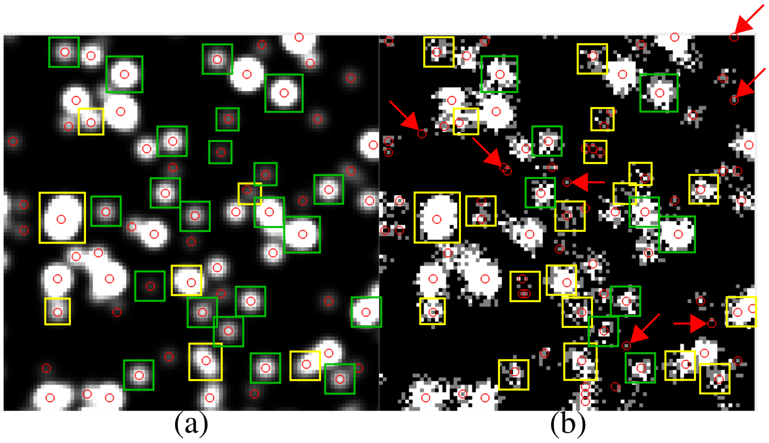

(a) (b)

Fig. S1. Result of the spot detection method used for cases (a) 3 (no noise, $t = 0.05$) and (b) 10 (Poisson noise, $t = 0.2$). Red circles are the centers of mass of detected spots. Green squares surround true spots without spot shift *i.e.* for which the closest detected spot position is exactly the true spot position. Yellow squares surround true spots with spot shift *i.e.* for which the closest detected spot position is not exactly the true spot position. In (b) red arrows point at detected spots positions which are not exactly on a true one *and* which are due only to the noise.

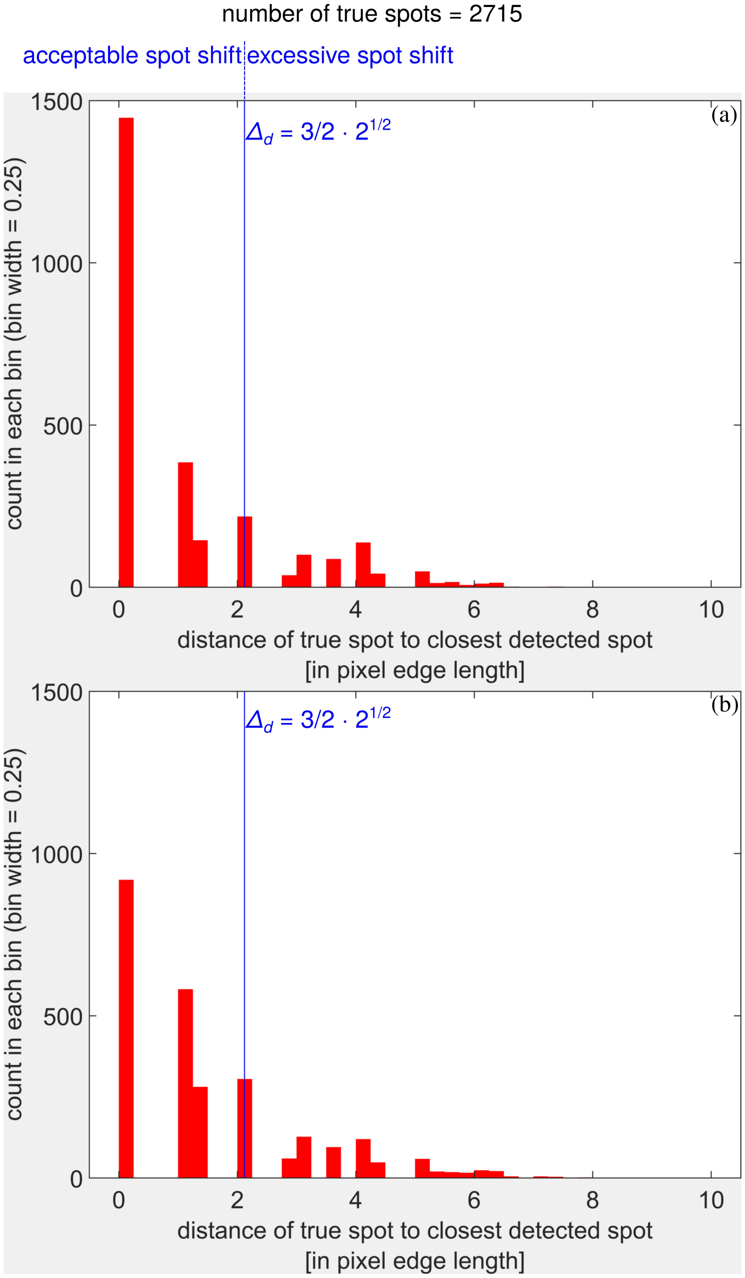

Fig. S2. Distribution of the spot shift (for each true spot, distance to the closest detected spot) in pixel edge length for cases (a) 3 (no noise, $t = 0.05$) and (b) 10 (Poisson noise, $t = 0.2$), in count mode (bar height = number of occurrences in the class). The chosen uncertainty Δ_d on the spot position on the detector demarcates the domains of acceptable and excessive spot shift.

S3. Expression of the calculation time and memory requirements

The expression of the time factor f in equation 5 comes from the following reasoning. First, the factor $\mathtt{n}^N$ can be explained as follows. Each candidate is constructed by taking one possible match among detected spots for each one of the N expected reflections. The number of possible matches among detected spots for each of the N expected reflections will be proportional to the number of spots, so the number of candidates to construct is proportional to $\mathtt{n}^N$. Before the construction process previously described, N expected reflections must be designated. Hence, all the choices of N expected reflections among the N_{pm} ones which have at least one possible match must be considered. Then N_{pm} can be approximated by $N + N^*$ the number of tested expected reflections, especially when the number of spots increases, because each tested expected reflection becomes more likely to have at least one possible match (even if it is a wrong one). Hence there will be N choices among $N + N^*$ *i.e.* $\frac{(N + N^*)!}{N! \cdot N^*!}$ choices, for each one of which the construction of all candidates will be performed in a time proportional to $\mathtt{n}^N$.

References

Dectris, (2021). Pilatus 3R for Laboratory - Dectris. https://www.dectris.com/products/pilatus3/pilatus3-r-for-laboratory/pilatus3-r-300k. Accessed January 18th, 2021.

Online Dictionary of Crystallography, (2021). Lorentz-polarization correction. https://dictionary.iucr.org/Lorentz%E2%80%93polarization_correction. Accessed January 18th, 2021.

Wolfram Research, Inc., (2021). Elementdata – wolfram language documentation. https://reference.wolfram.com/language/ref/ElementData.html. Accessed January 18th, 2021.
